# Multisensory Stimulation to Improve Low- and Higher-Level Sensory Deficits after Stroke: A Systematic Review

**DOI:** 10.1007/s11065-015-9301-1

**Published:** 2015-10-21

**Authors:** Angelica Maria Tinga, Johanna Maria Augusta Visser-Meily, Maarten Jeroen van der Smagt, Stefan Van der Stigchel, Raymond van Ee, Tanja Cornelia Wilhelmina Nijboer

**Affiliations:** Department of Experimental Psychology, Hemholtz Institute, Utrecht University, Utrecht, The Netherlands; Brain Center Rudolf Magnus, and Center of Excellence for Rehabilitation Medicine, University Medical Center Utrecht, and De Hoogstraat Rehabilitation, Utrecht, The Netherlands; Department of Brain, Body & Behavior, Philips Research Laboratories, Eindhoven, The Netherlands; Department of Biophysics, Donders Institute, Radboud University, Nijmegen, The Netherlands; Laboratory of Experimental Psychology, University of Leuven, Leuven, Belgium

**Keywords:** Stroke, Hemianopia, Perceptual disorders, Neglect, Rehabilitation, Multisensory, Review

## Abstract

The aim of this systematic review was to integrate and assess evidence for the effectiveness of multisensory stimulation (i.e., stimulating at least two of the following sensory systems: visual, auditory, and somatosensory) as a possible rehabilitation method after stroke. Evidence was considered with a focus on low-level, perceptual (visual, auditory and somatosensory deficits), as well as higher-level, cognitive, sensory deficits. We referred to the electronic databases Scopus and PubMed to search for articles that were published before May 2015. Studies were included which evaluated the effects of multisensory stimulation on patients with low- or higher-level sensory deficits caused by stroke. Twenty-one studies were included in this review and the quality of these studies was assessed (based on eight elements: randomization, inclusion of control patient group, blinding of participants, blinding of researchers, follow-up, group size, reporting effect sizes, and reporting time post-stroke). Twenty of the twenty-one included studies demonstrate beneficial effects on low- and/or higher-level sensory deficits after stroke. Notwithstanding these beneficial effects, the quality of the studies is insufficient for valid conclusion that multisensory stimulation can be successfully applied as an effective intervention. A valuable and necessary next step would be to set up well-designed randomized controlled trials to examine the effectiveness of multisensory stimulation as an intervention for low- and/or higher-level sensory deficits after stroke. Finally, we consider the potential mechanisms of multisensory stimulation for rehabilitation to guide this future research.

## Introduction

In the last decade there has been a considerable increase in fundamental cognitive neuroscience studies on multisensory integration (MSI; Van der Stoep et al. 2015). Most of these studies indicate that multisensory integration allows for a coherent representation of the environment and that it enhances detection and localization of external events (see Stein 2012 for an overview). Combining information from different sensory modalities can be especially beneficial in supporting behavior when the signal from a single modality is only weakly able to induce a behavioral response, or when a sensory system as a whole is weakened (Stein 2012). Based on these findings, we hypothesize that stimulating multiple sensory modalities (i.e., multisensory stimulation) has the potential to be beneficial in improving sensory deficits after brain damage, as information from a normally functioning sensory modality might aid the processing of information from the impaired sensory modality. In this way, multisensory stimulation might have the potential to aid rehabilitation of patients suffering from stroke.

Integration of multisensory information is an important aspect of multisensory stimulation. Animal studies have led to the formulation of three fundamental rules of MSI (Stein 2012): first, the *temporal rule* states that maximal MSI occurs when multimodal stimulations occur approximately at the same time; second, the *spatial rule* states that maximal MSI occurs when multimodal stimulations originate from the same location; and third, the *rule of inverse effectiveness* states that maximal MSI occurs when each of the constituent unisensory stimuli are suboptimally effective in evoking responses.

**Fig. 1:**
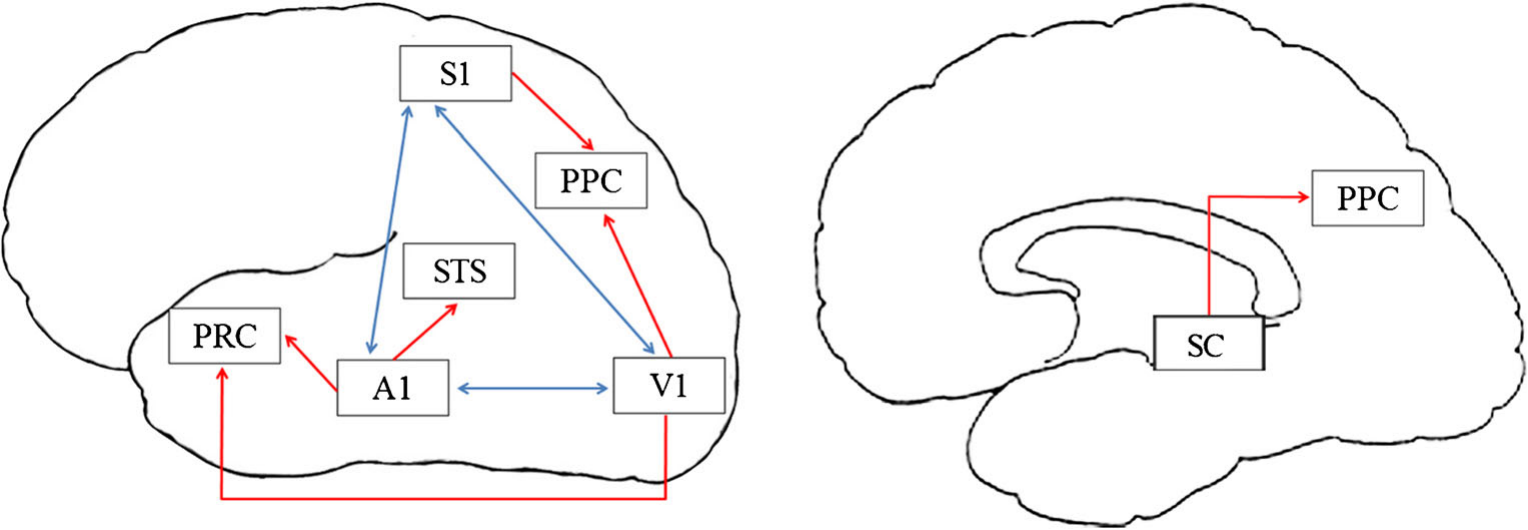
An illustration of how multisensory processing could arise from projections from sensory specific areas to multisensory convergence areas (depicted in *red*) or from direct anatomical connections between the primary sensory areas (depicted in *blue*). Lateral view on the left, medial view on the right. Depicted multimodal areas: The posterior parietal cortex (PPC); the superior temporal sulcus (STS) the perirhinal cortex (PRC); and the superior colliculus (SC). Depicted primary sensory areas: primary somatosensory (S1), visual (V1), and auditory (A1) cortex. See text for details

Electrophysiological and anatomical findings in animals and non-invasive neuroimaging findings in humans have identified multiple brain areas that contribute to MSI (Amedi et al. 2001; Keysers et al. 2003; Rockland and Ojima 2003; Shore 2005; Nagy et al. 2006; Beauchamp et al. 2008; Allman et al. 2009; Cappe et al. 2009; Falchier et al. 2010). Two basic neural mechanisms by which multisensory processing can arise have been proposed. First, multisensory processing may be accomplished when primary sensory areas are activated and project to multisensory convergence areas (red arrows in Fig. 1), followed by feedback projections from the latter to the former. Second, neurophysiological studies in animals have demonstrated that there is a direct neural connectivity between the primary sensory cortices (Rockland and Ojima 2003; Allman et al. 2009; Falchier et al. 2010), which implies that sensory modalities can also modulate each other’s responses at a low cortical level of processing (blue arrows in Fig. 1). Moreover, several fMRI (functional magnetic resonance imaging) studies have reported an increase or decrease in brain activity of the primary sensory cortices during multisensory stimulation (Macaluso et al. 2000; Amedi et al. 2002; Watkins et al. 2006; Martuzzi et al. 2007). For a more detailed discussion of brain areas that contribute to MSI, see for example Bolognini et al. (2013) and Klemen and Chambers (2012).

In general, the brain can use alternative routes to by-pass a damaged area after stroke and in this way adapt to the damage (e.g., Nudo et al. 1996; Dancause et al. 2005; Wilde et al. 2012; Buma et al. 2013). We expect that multisensory information could still be combined to some extent in the case of damage to multimodal association areas as well as when the damage affects sensory-specific cortices, because many (even sensory-specific) brain regions would still be able to assist in combining this information. Multisensory stimulation might even enhance residual neuronal activity within such a damaged area when information comes from multiple senses. This increase in neuronal activity might lead to (for instance) detection improvements, since neuronal activity is more likely to exceed the threshold necessary for detection. All in all, multisensory stimulation might prove to be a promising intervention for (sensory-specific) impairment caused by stroke, since information coming from multiple senses might enhance detection and localization of, and responding to external events, resulting in a reduction of the impairment.

The aim of the current systematic review is to provide an integrated account and quality assessment of studies that have investigated multisensory stimulation as a possible rehabilitation method to improve low-level and higher-level sensory deficits after stroke. Deficits in low-level processing of perceptual information occur at a relatively early processing stage, leading to a primary sensory deficit (e.g., visual field defects). Distortions at a later level of perceptual processing are causing higher-level sensory deficits, which are more cognitive in nature (e.g., neglect; Kandel et al. 2000). Recently, Johansson (2012) deemed multisensory stimulation in stroke rehabilitation a promising approach with a focus on *motor* recovery. Our focus will be on the effects of multisensory stimulation on recovery of *sensory* deficits. To guide future research, we also consider the mechanisms of multisensory stimulation for rehabilitation (i.e., the short- and long-term effects, transfer effects, and whether it targets compensation and/or restoration). In the next sections, studies that have assessed the effects of multisensory stimulation in patients with low-level visual (i.e., visual field defects), auditory and somatosensory deficits and higher-level sensory deficits (i.e., hemi-inattention or neglect) caused by stroke will be reviewed.

## Methods

### Literature Search and Article Selection

**Fig. 2:**
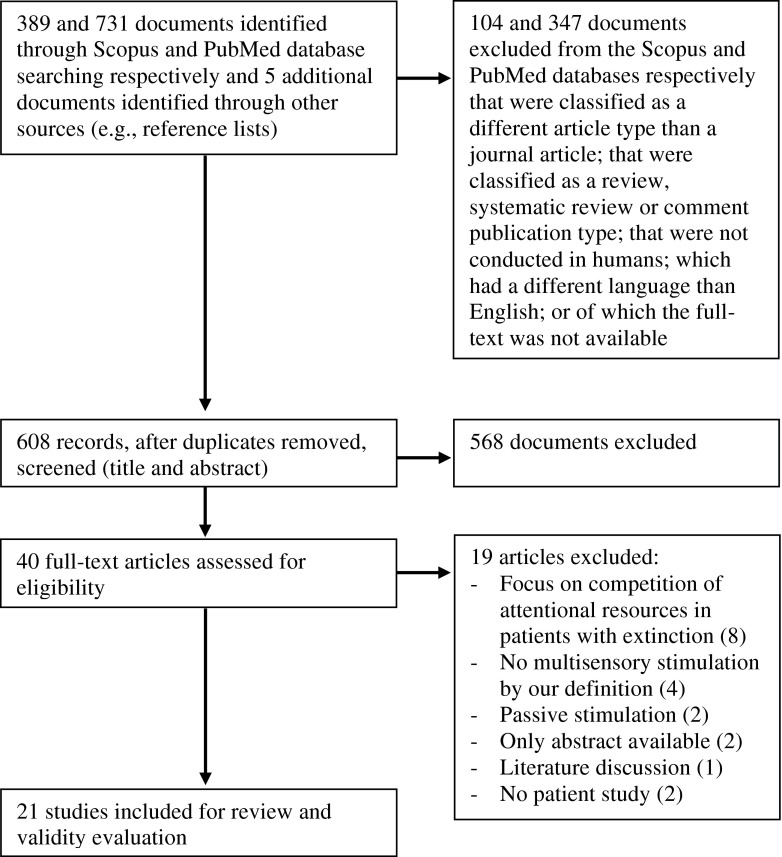
Schematic of the literature search and article selection used by the authors to identify studies on multisensory stimulation in stroke patients

The literature search (Fig. 2) was conducted in the Scopus and PubMed databases for articles that have been published before May 2015. Date last searched was May 5, 2015. The string used to search for articles was: (TITLE-ABS-KEY (“multisensory” or “multimodal integration” or “multimodal stimul*” or “audiovisual” or “audio-visual” or “visuo-auditory” or “visuotactile” or “visuo-tactile” or “tactile-visual” or “audiotactile” or “audio-tactile” or “tactile-audio” or “visual* enhanc*” or “tactile enhanc*” or “audit* enhanc*” or “somatosens* enhanc*”) AND (TITLE-ABS-KEY (“hemianop*” or “visual field defect” or “visual field deficit” or “auditory disorder” or “auditory deficit” or “auditory defect” or “somatosensory disorder” or “somatosensory defect” or “somatosensory deficit” or “perceptual disorder” or “perceptual deficit” or “perceptual defect” or “neglect” or “stroke”)) AND NOT (TITLE-ABS-KEY (“migraine” or “synesthesia” or “synaesthesia” or “spinal cord injury” or “autism” or “aphasia” or “schizophrenia” or “dyslexia”)). Documents retrieved from this initial search that were classified as an article by Scopus or as a journal article by PubMed and as being written in English were screened on their titles and abstracts. Studies were included which evaluated the effects of multisensory stimulation on patients with low- or higher-level sensory deficits caused by stroke. In all included studies at least two sensory modalities were stimulated at the exact same moment in time. The stimulation had to be passive and not active (i.e., the stimulation itself had to be independent of any action by the patient). Excluded were animal studies, studies in healthy participants (e.g., Laurienti et al. 2004; van Ee et al. 2009), reviews, and studies of which the full article was not available. Articles that focused on competition of multisensory attention in patients with extinction were excluded as well. The included articles were read completely and their references were scanned for relevant articles that might also meet criteria for inclusion. In total, 21 articles met the criteria for eligibility and were included for review and quality assessment.

### Quality Assessment

The quality of the included studies was assessed based on the following eight elements (following Spreij et al. 2014): 1) randomization; 2) inclusion of control patient group; 3) blinding of participants; 4) blinding of researchers; 5) follow-up (i.e., subsequent examination of participants); 6) group size; 7) reporting effect sizes; and 8) reporting time post-stroke. Studies could score 1 or 0 on each element, when it was dealt with in a sufficient or insufficient way respectively. Additionally, an element was scored as 0 if it could not be inferred from the article. If these quality elements were not sufficiently dealt with, the effect of an intervention might have been either under- or overestimated (Tijssen and Assendelft 2008).

The criteria for sufficient *randomization* were randomized allocation to an intervention or randomized or counterbalanced presentation of the order of conditions. *Inclusion of control patient group* was sufficient if a control group of patients receiving either an alternative form of treatment or no intervention was included. When patients and researchers were prevented from having access to certain information that might have influenced them and thereby the results, the criteria for *blinding of participants* and *blinding of researchers* respectively were sufficiently dealt with. The criteria for sufficient *follow*-*up* were incorporation of a follow-up in the study’s design and disclosure of the total number of losses-to-follow-up (i.e., dropouts). The element *group size* was scored as 1 when 10 or more patients were included in a within-subjects design or when 10 or more patients were included in each group in a between-subject design (this criterion is based on the common group size in fundamental studies in healthy participants [10–12 participants] and is used in other reviews as well [e.g., Spreij et al. 2014]). Additionally, *reporting effect sizes* and *reporting time post*-*stroke* were sufficiently dealt with when effect sizes and time post-stroke respectively were reported. If none of the elements were sufficiently dealt with, the study would receive a total score of 0, if all of the elements were sufficiently dealt with, the study would receive a total score of 8. Based on the study’s total score, its quality was classified as high (total score ≥ 6), moderate (total score ≥ 3 and ≤ 5), or low (total score ≤ 2).

## Results

The specifics of the included studies are presented in Tables 1–4. First, findings in patients with low-level, perceptual deficits are addressed, including patients with visual field defects (Table 1), auditory deficits (Table 2) and somatosensory deficits (Table 3). Second, findings in patients with neglect are addressed (Table 4).

**Table 1:** Studies evaluating the effects of multisensory stimulation after stroke in patients with visual field defects (in order of appearance in text)

Study	Patients	Lesion side	Lesion site	Time post injury	Stimulated modalities	Stimuli	Conditions	Frequency of stimulation	Outcome measure	Statistical test / alpha level	Main results
Frassinetti et al. 2005	7 neglect, 7 hemianopia, 7 neglect and hemianopia	Different across patients	Fr, Te, Pa, Oc, SC	?	V and A	V: single LED flash of 100 ms A: white-noise bursts of 100 ms	V, A, AV (stimuli presented temporally coincident, in same or different position)	2 sessions of +/− 2 h, including all conditions, on 2 succeeding d	V detection	ANOVA / *p* < .001	Sign. enhancement of V detection in the affected hemifield in AV condition when spatially coincident, in patients with neglect or hemianopia
Leo et al. 2008	12 hemianopia	7 RH, 4 LH, 1 both LH and RH	Fr, Te, Pa, Oc, SC	2 m – 30 y	A and V	A: pure-tone bursts of 100 ms V: squares presented for 100 ms	A, V, AV (stimuli presented temporally coincident, in same or different position)	15 blocks of 40 trials including all conditions, on 2 succeeding d	A localization	ANOVA / *p* < .03	Sign. improvement of A localization in the affected and unaffected hemifield in AV condition when spatially coincident (and temporally coincident, as demonstrated by another experiment in this study)
Ten Brink et al. 2015	7 hemianopia, 1 quadrantanopia	4 RH, 4 LH	Te, Pa, Oc, SC	26–154 m	A and V	A: broadband noise bursts of 500 ms (36–72 dB) V: circles presented for 500 ms	A, AV (stimuli presented temporally coincident, in same or different position)	Experiment 1: 40 blocks of 40 trials including all conditions Experiment 2: 2 blocks of 480 trials, first including A and AV coincident, second including A and AV disparate	Saccade accuracy (to A target) and latency (of initiation)	*t*-tests at single- subject level / *p* ≤ .004	Unaffected hemifield: sign. enhancement of saccade accuracy in AV coincident condition and sign. decrease of saccade accuracy in AV disparate condition (especially with high contrast V stimuli); sign. effects of condition on saccade latency for some patients Affected field: sign. enhancement of saccade accuracy in AV coincident condition for one patient
Cecere et al. 2014	1 central field defect and visual agnosia	Both LH and RH	Pa, Oc	3 y	V and A	V: solid lines presented for 250 ms A: looming (rising in intensity); receding (decreasing in intensity); or stationary (fixed intensity) sound of 250 ms	V, AV (A either looming, receding or stationary presented at same moment in time in either same or different position [also in different position in experiment 1])	Experiment 1: 40 blocks of 96 trials including all conditions Experiment 2: 40 blocks of 112 trials	Experiment 1: V discrimination Experiment 2: V detection	Fisher’s test / *p* < .038	V discrimination sensitivity (*d*') in the unaffected visual field was sign. higher in AV looming condition; V detection *d*' in the unaffected and affected visual field was sign. higher in all AV spatially coincident conditions
Brown et al. 2008	2 hemianopia	2 RH	Te, Pa, Oc	9 y and 32 y	V and P	V: 6 plexiglass block objects of 6 different sizes P: contralesional hand placed near or far from target location	Contralesional hand placed near or far from target location	The trials for each combination of V and P stimuli were repeated 6 times	Size estimation and grasping	Linear regression / *p* ≤. 047	Sign. enhancement of size estimation and grasping of objects presented in the left (affected) visual field in the hand-near condition
Schendel and Robertson 2004	1 hemianopia	1 RH	Te, Oc, SC	7 m	V and P	V: probe of 150 ms presented at 60 cm (baseline and near condition) or at 180 cm distance (far and tool condition) P: arm in lap or arm extended with or without holding a tennis racket	Contralesional arm in lap (baseline), arm extended (near), arm extended and visual stimuli presented further away (far), arm extended and holding tennis racket and visual stimuli presented further away (tool)	6 sessions conducted on multiple d	V detection	Chi-square test / *p* < .01	Sign. improvement of V detection in the left (affected) visual field in near condition compared to baseline. Sign. improvement in tool condition compared to far condition for the upper left visual field (after a correction for false alarms)
Smith et al. 2008	5 hemianopia	4 RH, 1 LH	Pa, Oc, SC	3.5–32 m	V and P	V: white spot (on black background) P: contralesional arm in lap or extended	Contralesional arm in lap (baseline) or extended (near)	Patients completed a different total amount of trials ranging from 96 to 240. Trials were divided in blocks	V detection	ANOVA / *p* ≤ .595 (and analysis for each individual patient with Fisher’s exact or Chi-square test / *p* ≤ .85)	No sign. differences between conditions (not for a single patient)
Passamonti et al. 2009a	9 hemianopia, 6 neglect	6 RH, 9 LH (neglect: all RH)	Fr, Te, Pa, Oc, SC	5–108 m	A and V	A: white-noise burst of 100 ms V: single LED flash of 100 ms	AV adaptation in which the stimuli were either spatially disparate of spatially congruent	Adaptation blocks lasted 4 min, A localization task had 105 trials	A localization before and after adaptation and A localization shift (calculated by subtracting mean reported locations pre-adaptation from those post-adaptation)	ANOVA / *p* < .05	After adaptation to spatial disparity: A localization accuracy decreased sign. after adapting the unaffected field; sign. shift in sound localization toward stimulation location; sign. greater shift in sound localization towards adapting stimulus after adaptation in the unaffected than the affected field in hemianopia patients. After adaptation to spatial coincidence: A localization accuracy sign. increased, regardless of the adapted hemifield; sign. greater accuracy for sounds presented at the adapted location compared to the untrained locations
Bolognini et al. 2005a	8 hemianopia	4 RH, 3 LH, 1 ?	Fr, Te, Pa, Oc	2–4 y	V and A	V: single LED flash of 100 ms A: white-noise burst of 100 ms	V, A, AV (stimuli presented at same or different location). The temporal interval in the audiovisual condition was gradually reduced from 500 to 0 ms	48 trials per block, total number of blocks differed across patients. Training was conducted on multiple daily sessions of +/− 4 h lasting less than 2 w	V detection (with and without eye movements), V exploration, hemianopic dyslexia and ADL assessed before training and at the end of the training and after 1 m	ANOVA / Wilcoxon signed-rank, *p* < .06	Improvements were demonstrated for the affected hemifield: V detection performance during training improved progressively. V detection was improved post-training, this improvement was sign. in the eye movement condition. V exploration was improved post-training for visual search and for the Number test (multiple sign. effects were found). Sign. improvements were also demonstrated for hemianopic dyslexia and ADL
Passamonti et al. 2009b	12 hemianopia and 12 healthy controls	5 RH, 5 LH, 2 ?	Fr, Te, Pa, Oc, SC	5 m – 30 y	V and A	V: single LED flash of 100 ms A: white-noise burst of 100 ms	V and AV training. The temporal interval in the AV training was gradually reduced from 300 to 0 ms	Training was conducted on multiple daily sessions of +/− 4 h lasting less than 2 w	V detection (with and without eye movements), V exploration, ADL, and occulomotor scanning assessed before training, after V training, after AV training, 3 m later and 1 y later	ANOVA / *p* < .05	After the AV training patients improved sign. in V detections, perceptual sensitivity and ADL. In addition, after the AV training, patients demonstrated sign. fewer fixations and a sign. reduction in mean saccadic amplitude. As a consequence, V scanning was more organized and more similar to the control subjects. Training effects in patients remained stable at the 3-month and the 1-year follow-up for V detection and exploration, oculomotor scanning and ADL
Keller and Lefin-Rank 2010	13 hemianopia and 7 quadrantanopia	Quadrantanopia: 6 RH, 1 LH. Hemianopia: 7 RH, 6 LH	Te, Pa, Oc	3–24 w	V and A	V: single LED flash of 100 ms A: white-noise burst of 100 ms	V and AV training	20 sessions of each 30 min over 3 w	V exploration (for reading and object search), occulomotor scanning and ADL assessed before and after training	ANOVA / *p* ≤ .036	Patients receiving AV training improved sign. more on all outcome measures

**Table 2:** Studies evaluating the effects of multisensory stimulation after stroke in patients with auditory deficits (in order of appearance in text)

Study	Patients	Lesion side	Lesion site	Time post injury	Stimulated modalities	Stimuli	Conditions	Frequency of stimulation	Outcome measure	Statistical test / alpha level	Main results
Bolognini et al. 2005b	1 auditory localization defect	1 RH (temporal-occipital)	Te, Oc	9 m	A and V	A: white-noise burst of 100 ms V: single LED flash of 100 ms	A, V, AV (stimuli presented in same position or in different position)	120 A, 120 V, 120 AV spatially coincident and 360 AV spatially disparate trials. Trials were distributed in 15 experimental blocks over 3 consecutive d	Percentage of correct responses of A localization	ANOVA / *p* < .0005	Sign. improvement of A localization in the AV condition when the stimuli were spatially coincident

**Table 3:** Studies evaluating the effects of multisensory stimulation after stroke in patients with somatosensory deficits (in order of appearance in text)

Study	Patients	Lesion side	Lesion site	Time post injury	Stimulated modalities	Stimuli	Conditions	Frequency of stimulation	Outcome measure	Statistical test / alpha level	Main results
Newport et al. 2001	1 somatosensory deficit of the right upper limb and 1 healthy control	1 LH	SC	+/− 3 y	P and V	P: index finger of unseen target hand, placed underneath a surface V: target locations defined by small wooden pin (V condition) or viewing surface adjacent to hidden limb	V, P (in which adjacent surface to the to-be-detected limb could not be seen) and VP (in which adjacent surface could be seen)	32 trials for each hand in each condition (total = 192 trials). Patient was tested in 2 sessions	Localization of the target (by pointing, to a V stimulus in the V condition and to the unseen limb in the other 2 conditions)	ANOVA / *p* < .0001	For the patient, detection of the impaired hand was sign. improved when the adjacent surface could be seen (VP condition)
Serino et al. 2007	10 somatosensory deficit and 32 healthy controls	5 RH, 5 LH	Fr, Te, Pa, SC	1–50 m	S and V	S: 1 or 2 vibrating solenoids attached to the underarm (2: separated by 30–90 mm) V: viewing either the own arm, a neutral object or a rubber foot	Viewing own arm, viewing a neutral object and viewing a rubber foot	24 single taps, 24 simultaneous double taps	Two point discrimination (tactile acuity)	ANOVA / *p* < .03 (and linear regression and ANOVA on the healthy control data, *p* < .04)	Performance in patients (and in subjects with low tactile accuracy) was sign. enhanced when own arm was viewed

**Table 4:** Studies evaluating the effects of multisensory stimulation after stroke in patients with neglect (in order of appearance in text)

Study	Patients	Lesion side	Lesion site	Time post injury	Stimulated modalities	Stimuli	Conditions	Frequency of stimulation	Outcome measure	Statistical test	Main results
Calamaro et al. 1995	8 neglect, 7 without neglect and 8 healthy controls	15 RH	Fr, Te, Pa, Oc, SC	at least 1 m (not completely clear)	A and V	A: consonant-vowels V: dummy loudspeaker	A stimulation on the left or right without (baseline) and with (experimental) spatially congruent or incongruent V stimulation	36 baseline trials, 72 experimental trials	A identification	ANOVA / *p* < .01	No difference in identification of left A stimulation between neglect patients and controls when dummy speaker was presented on the right side
Soroker et al. 1995	7 neglect (including auditory neglect) and 8 healthy controls	7 RH	Fr, Te, Pa, Oc, SC	?	A and V	A: consonant-vowels V: movie of pronounced syllables	A stimulation, AV stimulation (in which A stimuli were presented in contralesional space and V stimuli in ipsilesional space)	Max. 36 trials for each stimulation	A identification	ANOVA and *t*-test / *p* ≤ .038	AV stimulation increased A identification in both patients and controls. Improvement was bigger when V stimulation had a low saliency (i.e., slight lip opening; compared to high saliency) and when V stimulation was congruent to the A stimulation (compared to incongruent)
Passamonti et al. 2009a	9 hemianopia, 6 neglect	6 RH, 9 LH (neglect: all RH)	Fr, Te, Pa, Oc, SC	5–108 m	A and V	A: white-noise burst of 100 ms V: single LED flash of 100 ms	AV adaptation in which the stimuli were either spatially disparate of spatially congruent	Adaptation blocks lasted 4 min, A localization task had 105 trials	A localization before and after adaptation and A localization shift (calculated by subtracting mean reported locations pre-adaptation from those post-adaptation)	ANOVA / *p* < .05	After adaptation to spatial disparity: A localization accuracy decreased sign. after adapting the normal field; sign. shift in sound localization toward stimulation location; sign. greater shift in sound localization towards adapting stimulus after adaptation in the normal field than the affected field in hemianopia patients. After adaptation to spatial coincidence: A localization accuracy sign. increased, regardless of the adapted hemifield; sign. greater accuracy for sounds presented at the adapted location compared to the untrained locations
Frassinetti et al. 2002	7 neglect and 8 healthy controls	7 RH	Fr, Te, Pa, SC	1 m – 14 y	V and A	V: single LED flash of 100 ms A: pure tones of 150 ms	V, A, AV (stimuli presented in same or in different position)	8 trials per condition, run in 2 sessions of +/− 1 h on consecutive d	V detection	ANOVA / *p* < .05	Performance in the left V field was sign. enhanced in the AV condition (mostly when the stimuli were spatially coincident)
Frassinetti et al. 2005	7 neglect, 7 hemianopia, 7 neglect and hemianopia	Different across patients	Fr, Te, Pa, Oc, SC	?	V and A	V: single LED flash of 100 ms A: white-noise bursts of 100 ms	V, A, AV (stimuli presented temporally coincident, in same or different position)	2 sessions of +/− 2 h, including all conditions, on 2 succeeding d	V detection	ANOVA / *p* < .001	Sign. enhancement of V detection in the affected hemifield in AV condition when spatially coincident, in patients with neglect or hemianopia
van Vleet and Robertson 2006	1 neglect	1 RH	Fr, Te, Pa, SC	8 w	V and A	V: target among distractors (sharing either color or shape feature with target) A: tone of 2000 ms (presented at onset of V search array, congruent or incongruent to V target location)	No A stimulation, bilateral sound, one-sided spatially congruent sound, one-sided spatially incongruent sound (tone was not predictive of target location)	34 trials for each condition tested over 4 sessions	V search efficiency (presentation latency in a conjunction V search task with a 75 % accurate target detection)	ANOVA / *p* < .01	Search efficiency for targets in the impaired hemifield was sign. increased in the sound conditions, when compared to the no-sound condition. In addition, improvement in the spatially congruent sound condition was larger than in the other sound conditions
Làdavas et al. 1997	29 patients with RH damage of which 20 with and 9 without neglect	29 RH	Fr, Te, Pa, Oc, SC	0.5–12 m	V and P	V: 28 line drawings presented left or right, 2 in center. 10 distractor drawings presented left or right, 2 in center. All viewed via mirror P: left or right hand passively moved in left, right or center space	Left or right hand passively moved in left, right or center space for duration of trial. The passively moved hand was seen only in the mirror (and thus viewed inverted) when in left or right space, but was not seen at all in center space	6 trials: 1 trial per condition in which the patient continued naming all target stimuli (the line drawings) until the patient stated that all targets had been named	V identification	ANOVA / *p* < .05	Performance of patients with neglect in naming line drawings on the left side of the mirror was sign. more accurate when the left hand was moved in the left than in the right or center space. There was no sign. effect for the right hand and for the right side of space. The patients without neglect showed no sign. effects
di Pellegrino and Frassinetti 2000	1 left-sided visual extinction (without neglect)	1 RH	Te, Pa	+/− 16 m	V and P	V: 1 digit presented on the right or left, 2 digits simultaneously presented on both sides P: index fingers placed on table top, or on screen surface, directly below target, while fingers were or were not occluded from view	Fingers far (index fingers aligned with target at 40 cm distance), fingers near (index fingers positioned on screen), fingers covered (identical to fingers near, but with fingers occluded from view), V cues (target at 40 cm distance, photographs of index fingers were displayed on the screen)	4 blocks of 18 trials for each condition, tested in 4 separate sessions	Extinction score (i.e., proportion correct identification in bilateral trials divided by proportion correct in unilateral trials)	ANOVA / *p* < .0008	Extinction score was less in fingers near condition than in the other conditions
Sambo et al. 2012	4 neglect with tactile extinction or somatosensory deficits and 8 healthy controls	4 RH	Fr, Te, Pa, Sc	1–14 m	S, P and V	S: single taps delivered with solenoids P: left hand placed either in left or right hemi-space V: vision of the left hand was either available or prevented	Left hand placed in left (contralesional) hemispace or in right (ipsilesional) hemispace with either vision or no vision of the left hand	320 trials equally distributed over 8 experimental blocks	T detection	ANOVA / *p* ≤ .043	Performance of patients was sign. faster when the left hand was placed in the right hemispace. In addition, this effect was sign. greater when the hand was visible. Healthy controls were sign. faster when the left hand was placed in the left hemispace and vision had no sign. effect for these participants

### Visual Field Defects

Visual field defects, such as hemianopia, occur frequently after stroke, as a result of a lesion in the early visual pathway (Kandel et al. 2000). Patients with visual field defects usually fail to adequately respond to or report contralesional visual stimuli (Halligan et al. 2003), resulting in difficulties with reading, scanning scenes, and obstacle avoidance, especially on their affected side (Papageorgiou et al. 2007). Eleven studies were included that have examined direct and short-term effects (i.e., effects measured during stimulation or directly after stimulation) and/or short-term effects and longer lasting effects (i.e., effects measured not directly after stimulation) of multisensory stimulation on performance of patients with chronic and acute visual field defects. The characteristics of the studies are listed in Table 1.

Studies on the direct and short-term effects of multisensory stimulation by Frassinetti et al. (2005) and Leo et al. (2008) demonstrated that the addition of a coincident sound enhanced detection of a visual target in the affected hemifield (Frassinetti et al. 2005) and vice versa (Leo et al. 2008). In a recent study by Ten Brink et al. (2015) the addition of a coincident sound facilitated saccades to a visual target in the unaffected hemifield of all (eight) patients, but in only one patient performance in the affected hemifield was enhanced. In addition, Cecere et al. (2014) presented a patient with a complete loss of central vision and visual agnosia with different types of sounds (looming, receding, and stationary) while examining his visual discrimination and detection abilities. Visual *discrimination* in the unaffected, but not affected, visual field was enhanced by the addition of a coincident looming sound. Visual *detection* was enhanced by the addition of all types of coincident sounds in both the unaffected and affected field.

Brown et al. (2008) demonstrated that proprioceptive information provided by placing patients’ left hand near objects improved target size processing of these objects in the hemianopic field. Likewise, in a single case study by Schendel and Robertson (2004), visual detection in the affected hemifield improved when the patient’s contralesional arm was extended into the affected field, but only for visual stimuli near the extended hand. However, these findings could not be replicated in a sample of five patients in an otherwise largely similar study of Smith et al. (2008).

Apart from the direct and short-term improvements, longer lasting effects have also been reported. Passamonti et al. (2009a) found that auditory localization improved after four minutes of adaptation to spatially congruent audiovisual stimulation, especially at the location where audiovisual stimuli were presented. Bolognini et al. (2005a) presented patients with unisensory and multisensory trials in daily sessions of about four hours for nearly two weeks. Patients improved in visual detection, visual exploration and in different tasks of daily life (relating to visual impairments), and these improvements were stable at the follow-up after one month. This study, however, did not rule out that a similar improvement might have been obtained by only using unisensory (visual) stimulation, as all the different conditions were incorporated in the sessions. To overcome this confound, Passamonti et al. (2009b) incorporated an unisensory, visual training as well as an audio-visual training and showed that audiovisual training improved visual detection and exploration, oculomotor scanning and activities of daily life, whereas the visual training did not. These effects remained stable at a three-month follow-up and a one-year follow-up.

Keller and Lefin-Rank (2010) examined the effects of audiovisual stimulation in patients in the subacute stage after brain damage. Patients received either audiovisual training or visual training. The audiovisual training resulted in a larger improvement in visual exploration compared to the visual training. In addition, only patients that had received audiovisual training showed near normal daily living activities (relating to visual impairments) after the training of three weeks. Yet, the role of spontaneous recovery in these findings is not clear, as there was no group of patients receiving no training at all.

### Auditory Localization Deficits

Only a single study examining the effects of multisensory stimulation on specific auditory deficits after stroke was included (Table 2). Bolognini et al. (2005b) investigated whether a temporally congruent visual stimulus improved the localization of an auditory stimulus in a patient with a selective deficit of auditory spatial localization, yet intact detection, in the whole auditory field. Auditory localization improved, but only when the visual stimulus was spatially congruent.

### Somatosensory Deficits

Impaired somatosensory function has negative effects on exploration of the environment, spontaneous use of hands, precision grip and object manipulation. Additionally, it has negative effects on rehabilitation outcomes, such as personal safety, functional outcome and quality of life (Carey 1995; Carey and Matyas 2011). Two studies on the effect of multisensory stimulation in patients with somatosensory deficits were included (Table 3). These studies examined the effect of viewing either a relevant body part or the surface adjacent to it. When a relevant body part or its adjacent surface is viewed, stimulation might be provided by descending modulatory inputs from visual body representation areas which could aid in the reorganization of damaged brain areas in somatosensory deficits.

Newport et al. (2001) investigated the effect of combining vision and proprioception in a patient with a unilateral somatosensory impairment of the right upper limb, including right tactile extinction (i.e., the failure to report a contralesional stimulus only when it is delivered together with a concurrent ipsilesional stimulus [Gallace and Spence 2008]). When the patient could view the surface adjacent to her hidden to-be-localized limb, detection of the impaired limb improved compared to when she was blindfolded. In addition, a single case study by Serino et al. (2007) indicated that during invisible stimulation of the upper limb, tactile thresholds were improved when the own upper limb was viewed compared to viewing a rubber foot or a neutral object.

### Neglect

Patients with unilateral spatial neglect suffer from impaired explicit spatial processing (i.e., reporting and/or exploring) of stimuli presented in the affected contralesional space (Gallace and Spence 2008; Ting et al. 2011). Additionally, patients with neglect can have a disrupted mental representation of space, which is generally shifted to the ipsilesional space and therefore underrepresents the contralesional space (Mesulam 1999; Zamarian et al. 2007). Effective rehabilitation of neglect is of utmost importance as the disorder is associated with poorer cognitive and motor recovery and poorer outcomes on ADL (activities of daily living; Heilman et al. 2000; Buxbaum et al. 2004; Nijboer et al. 2013, 2014). Neglect can occur in all perceptual domains (Kinsbourne 1993) and in different regions of space (Aimola et al. 2012; Van der Stoep et al. 2013). Extinction often occurs in patients with neglect, however, double dissociations have been reported (Pavlovskaya et al. 2007). Nine studies were included that have examined the effects of multisensory stimulation on performance of patients with neglect and/or extinction. The characteristics of these studies are listed in Table 4.

Two early studies of Calamaro et al. (1995) and Soroker et al. (1995) demonstrated that *identification* of auditory stimuli in the impaired hemispace of patients with neglect was enhanced with additional visual stimulation in the intact hemispace. Passamonti et al. (2009a) demonstrated that auditory *localization* in patients with neglect was improved after four minutes of adaptation to spatially congruent, but not spatially incongruent, audiovisual stimuli, especially at the adapted location.

Furthermore, as demonstrated in the studies of Frassinetti et al. (2002, 2005), detection of a visual stimulus improved on the contralesional side when a spatially congruent sound was presented simultaneously. van Vleet and Robertson (2006) demonstrated that target detection improved in the impaired hemifield when a tone was presented at the onset of the search display in a location congruent to the target location.

Làdavas et al. (1997) and di Pellegrino and Frassinetti (2000) examined whether position of the hands could modulate visual neglect or visual extinction respectively. In the first study, patients with neglect viewed visual targets and distractors via a mirror and were more accurate in identifying targets on the left side of the mirror when the left hand was passively moved on the left side of space (Làdavas et al. 1997). In the di Pellegrino and Frassinetti study (2000) a patient’s visual extinction for left targets was reduced when the patient’s fingers were positioned below the visual targets. The left-sided extinction was not reduced when the patient’s fingers were occluded from view.

Furthermore, Sambo et al. (2012) examined the effect of proprioceptive and visual information on processing of tactile stimuli in patients with both neglect and left tactile extinction or somatosensory deficits. Processing of left invisible tactile stimulation was enhanced when patients placed their left hand in the ipsilesional, ‘intact’, hemispace compared to when they placed the hand in the contralesional, ‘impaired’, hemispace, especially when patients were able to see the hand.

## Quality Assessment

In the section above the included studies on multisensory stimulation after stroke were discussed. Overall, twenty out of twenty-one studies reported a beneficial effect of multisensory stimulation in improving sensory deficits. In this section we assess the discussed studies on 1) randomization; 2) inclusion of control patient group; 3) blinding of participants; 4) blinding of researchers; 5) follow-up; 6) group size; 7) reporting effect sizes; and 8) reporting time post-stroke (Table 5).

**Table 5:** Scores of the quality assessment of the discussed studies, based on eight elements ^a^

Study	Randomization of intervention or conditions	Inclusion of control patient group	Blinding of participants	Blinding of researchers	Follow-up	Group size	Reporting effect sizes	Reporting time post-stroke	Total	Quality
Frassinetti et al. 2005	1	0	0	0	0	0	0	0	1	low
Leo et al. 2008	0	0	0	0	0	1	0	1	2	low
Ten Brink et al. 2015	0	0	0	0	0	0	0	1	1	low
Cecere et al. 2014	0	0	0	0	0	0	0	1	1	low
Brown et al. 2008	1	0	0	0	0	0	0	1	2	low
Schendel and Robertson 2004	1	0	0	0	0	0	0	1	2	low
Smith et al. 2008	1	0	0	0	0	0	0	1	2	low
Passamonti et al. 2009a	0	0	0	0	0	0	0	1	1	low
Bolognini et al. 2005a	0	0	0	0	1	0	0	1	2	low
Passamonti et al. 2009b	0	0	0	1	1	1	0	1	4	moderate
Keller and Lefin-Rank 2010	1	1	0	0	0	1	0	1	4	moderate
Bolognini et al. 2005b	1	0	0	0	0	0	0	1	2	low
Newport et al. 2001	1	0	0	0	0	0	0	1	2	low
Serino et al. 2007	1	0	0	0	0	1	0	1	3	moderate
Calamaro et al. 1995	0	0	0	0	0	0	0	0	0	low
Soroker et al. 1995	0	0	0	0	0	0	0	0	0	low
Frassinetti et al. 2002	1	0	0	0	0	0	0	1	2	low
van Vleet and Robertson 2006	1	0	0	1	0	0	0	1	3	moderate
Làdavas et al. 1997	1	0	0	0	0	1	0	1	3	moderate
di Pellegrino and Frassinetti 2000	1	0	0	0	0	0	0	1	2	low
Sambo et al. 2012	1	0	0	0	0	0	1	1	3	moderate

^a^ 0 = element was dealt with insufficiently; 1 = element was dealt with sufficiently

### Study Characteristics

Of the 21 discussed studies, only 1 (Keller and Lefin-Rank 2010) consisted of a between-subjects design. The other studies were within-subjects designs, 6 (di Pellegrino and Frassinetti 2000; Newport et al. 2001; Schendel and Robertson 2004; Bolognini et al. 2005b; van Vleet and Robertson 2006; Cecere et al. 2014) of which were single case studies. On a 8-point scale (representing the 8 elements on which the articles were assessed, with 8 indicating the highest and 0 the lowest possible score), the average total score for all studies was 2 (*SD* = 1.1, range 0–4). Based on the total scores, 6 studies were of moderate quality (4 studies had a total score of 3, and 2 studies had a total score of 4) and fifteen were of low quality (2 studies had a total score of 0, 4 studies had a total score of 1, and 9 studies had a total score of 2). The average score for studies on visual field defects was 2 (11 studies), on auditory deficits 2 (1 study), on somatosensory deficits 2.5 (2 studies), and on neglect 2 (9 studies). Of the 21 studies, only a single study (Smith et al. 2008), which included patients with hemianopia, did not report beneficial effects of multisensory stimulation, this study was assessed with a total score of 2.

All discussed studies included detection, localization, exploration, discrimination and/or identification outcome measures in their design. Only 3 of the 21 studies discussed (all on hemianopia; Bolognini et al. 2005a; Passamonti et al. 2009b; Keller and Lefin-Rank 2010) included ADL outcome measures in their design, rendering the discussed studies’ foci mostly experimental.

### Randomization and Inclusion of Control Group

Ideally, studies investigating the effect of an intervention should have a group of patients receiving an intervention and a control group of patients, either not receiving any intervention or receiving a ‘control intervention’ (Higgins et al. 2011). Only a single study (Keller and Lefin-Rank 2010) had incorporated a control patient group: two randomly allocated groups of patients with hemianopia or quadrantanopia received either multisensory or unisensory training. The study demonstrated that patients benefited more from multisensory training than unisensory training.

In all the other discussed studies, each patient participated in at least two different conditions, namely an experimental condition (with multisensory stimulation) and a control condition (without multisensory stimulation). In this way, effects of multisensory stimulation could be compared within patients. Twelve of twenty within-subjects design studies (Làdavas et al. 1997; di Pellegrino and Frassinetti 2000; Newport et al. 2001; Frassinetti et al. 2002; Schendel and Robertson 2004; Bolognini et al. 2005b; Frassinetti et al. 2005; van Vleet and Robertson 2006; Serino et al. 2007; Brown et al. 2008; Smith et al. 2008; Sambo et al. 2012) reported that they had randomized or counterbalanced the different conditions, in the other studies this was not reported.

To allow for monitoring of ‘practice effects’ and verification of improvement of performance toward normal levels, a control group of healthy participants is very useful. Only six of the studies incorporated a control group of healthy subjects (Calamaro et al. 1995; Soroker et al. 1995; Newport et al. 2001; Passamonti et al. 2009b; Serino et al. 2007; Sambo et al. 2012) but in one study (Passamonti et al. 2009b) the healthy control group did not receive the exact experimental training as the patients. These studies demonstrated that the effect of multisensory stimulation was larger in patients compared to healthy controls (Newport et al. 2001; Passamonti et al. 2009b; Sambo et al. 2012) or that patients could perform on the level of healthy controls when multisensory information was presented (Calamaro et al. 1995). Moreover, these studies demonstrated that multisensory stimulation could lead to improvements both in patients and healthy controls (Soroker et al. 1995) or in patients and healthy controls with a low sensory acuity only (Serino et al. 2007).

### Blinding of Participants and Researchers

Only three of the twenty-one studies reported that researchers were blinded for one important aspect (van Vleet and Robertson 2006; Passamonti et al. 2009b; Keller and Lefin-Rank 2010). Yet, in one of these studies (Keller and Lefin-Rank 2010) not all researchers were blinded. The two studies that dealt with blinding of researchers sufficiently demonstrated that multisensory stimulation could be beneficial in patients with hemianopia (Passamonti et al. 2009b) or neglect (van Vleet and Robertson 2006). None of the studies reported that patients were blinded. As a direct result of the design chosen, blinding is more difficult when each patient is tested in all conditions and when the difference between the conditions is clear (for example: performing a task with or without a distinctive sound), which was the case in most of the discussed studies.

### Follow-Up

Sufficient follow-up to the examination is of great importance to assess the effects of the intervention over a prolonged period of time. Only two of the twenty-one studies discussed (Bolognini et al. 2005a; Passamonti et al. 2009b) incorporated a follow-up in their design (with no losses-to-follow-up). They found that patients with hemianopia could improve in visual detection and (oculomotor) exploration in the impaired field and in different tasks of daily life with multisensory stimulation. These beneficial effects were stable at a one month (Bolognini et al. 2005a) and at a one year (Passamonti et al. 2009b) follow-up. With respect to the other studies on low-level sensory impairment and neglect, no follow-up results were reported.

### Group Size

Studies with small groups often have large confidence intervals and are less able to detect clinically relevant effects statistically. Group sizes in the discussed studies were relatively small. The average group size in the discussed studies was 6.36 (*SD* = 4.81, range 1–20). The largest group had 20 patients (Làdavas et al. 1997), 9 studies (including 6 case studies) had 1 to 5 patients, and 11 studies had 7 to 12 patients. The average group size for studies on visual field defects was 6.7 (11 studies), on auditory deficits 1 (1 study), on somatosensory deficits 5.5 (2 studies), on neglect 6.9 (9 studies). One of the twenty-one discussed studies (Smith et al. 2008) did not find a difference between unisensory and multisensory stimulation in patients with hemianopia; this study included 5 patients.

### Reporting Effect Sizes and Time Post-Stroke

An important factor that should be reported is the effect size to determine the strength of the statistically significant results. Yet, only 1 of the 21 studies discussed (Sambo et al. 2012) reported effect sizes. This study found that multisensory stimulation enhanced tactile detection, with an effect size (*η2*) ranging from 0.46 to 0.76, which is considered as a large effect size (Cohen 1988). Another important factor that should be mentioned is the time post-stroke onset to verify response to treatment in different phases of recovery. Three of the studies discussed (Frassinetti et al. 2005; Calamaro et al. 1995; Soroker et al. 1995) did not report the time post-stroke of their included patients. Overall, studies demonstrating beneficial effects of multisensory stimulation included patients between 0.5 months to 32 years post stroke, effects in the early acute phase were not reported.

## Discussion

This review has attempted to assess and integrate evidence for the effectiveness of multisensory stimulation as a possible rehabilitation method for functional recovery for patients with low- or higher-level sensory deficits after stroke. We hypothesized that multisensory stimulation has the potential to be beneficial for these groups of patients, as information from a normally functioning sensory modality might aid the processing of information from the impaired sensory modality. Twenty of the twenty-one included studies demonstrated beneficial effects of multisensory stimulation on patients with low- and/or higher-level sensory deficits. These studies demonstrated that detection, localization, exploration, discrimination, identification, and even several activities of daily living could be enhanced by multisensory stimulation in both patients with low- and patients with higher-level sensory impairments. Notwithstanding these beneficial effects, our quality assessment classified 6 studies as being of moderate and 15 studies as being of low quality. Most studies employed a within-subjects design with small groups and more than a third of these studies did not report taking into account randomization/counterbalancing of the different conditions. In addition, none of the studies reported blinding all important aspects, only two studies incorporated a follow-up in their design, three studies did not report time post-stroke, and only one study reported effect sizes. Most importantly, the discussed studies’ foci were mostly experimental (focusing on tasks such as signal detection or signal localization); only three studies measured the effect of multisensory stimulation on ADL-measures. Therefore, at present, none of the studies on multisensory stimulation after stroke are adequate to give a proper evaluation of the effectiveness of the method as an intervention. We believe that now is the time to take this line of research to the next level and set-up well-designed randomized controlled trials (RCTs), in which the important discussed quality elements are taken into account and in which ADL-measures are included, to examine the effectiveness of multisensory stimulation as an intervention after stroke.

### Starting Points for Future Research

Regarding the type of stimulation, a good starting point for an RCT might be audiovisual stimulation, as most included studies (13 out of 21) focused on this type of stimulation and this type of stimulation is well controllable. Hemianopia might then be a suitable candidate to target first, as most studies focused on patients with hemianopia (10 out of 21), this impairment occurs relatively frequently after stroke, and the implicated visual neural networks are well-documented (e.g., Kandel et al. 2000). Yet, recent research demonstrated that visual spatial localization can be distorted in patients with hemianopia (Fortenbaugh et al. 2015), which can complicate any spatial multisensory approach to rehabilitation. It is therefore essential that future studies in patients with hemianopia include appropriate baseline conditions to measure also these distortions. In this way, future studies can control for the influence of any comorbid (visual) disorders on effects of multisensory stimulation. After establishing an effective protocol for patients with hemianopia, the effectiveness of the protocol can be examined for other disorders as well. Expanding collaborations between fundamental and clinical researchers might ensure that potentially interesting techniques can be studied in clinical populations soon after fundamental studies demonstrate positive effects of these techniques.

### Potential Mechanisms for Rehabilitation

When considering multisensory stimulation as an intervention, it is of importance to determine if improvements might result from recovery or compensatory responses. Recovery is the reappearance of pre-stroke function and is characterized by restitution or repair of the functionality of damaged neural structures (Levin et al. 2009). Compensation, on the other hand, is the reduction of the disparity between an impaired function and the environmental demands characterized by activation in alternative brain areas that are not normally activated in controls (Levin et al. 2009).

While most studies demonstrated short-term beneficial effects of multisensory stimulation, two studies (Bolognini et al. 2005a; Passamonti et al. 2009b) provided evidence that effects of multisensory ‘training’ (of less than two weeks) can persist for a longer period of time (up to one year). The underlying mechanisms of these long-term effects, which are especially interesting from a rehabilitation point of view, are not yet established. To speculate, long-term effects might mainly result from *restoration*, as multisensory stimulation might recruit and, in turn, strengthen residual (sensory) pathways in the brain and thereby might restore sensory performance and function (Jiang et al. 2015). Direct effects, on the other hand, might mainly result from passive *compensation* (e.g., multisensory stimuli surpassing the attention threshold, thereby ‘normalizing’ sensory performance and function), as neurobiological recovery is known to require more time to complete (Teasell and Hussein 2014). Possibly, direct effects might mostly reflect enhanced attention to the stimulated location. It might be especially effective to target restoration in the first three months post-stroke, as in this period it is most likely that neurobiological recovery will take place (Kwakkel et al. 2004; Levin et al. 2009; Nijboer et al. 2013). Future studies should aim at identifying an optimal timing at which multisensory stimulation would be most effective.

Restoration of sensory performance and function might also lead to improvements on ADL (see Bolognini et al. 2005a; Passamonti et al. 2009b; Keller and Lefin-Rank 2010). Future studies should therefore examine the effects of restoration on cognitive (such as attention and memory) outcome measures, and to what extent the effects of multisensory stimulation are transferred (on the long-term). This could for example be achieved by assessing these outcomes before and after (with multiple follow-up measurements) multisensory ‘training’.

When considering multisensory stimulation as an intervention in future research, it is also essential to determine the optimal frequency, duration and intensity of multisensory stimulation and which patients benefit from multisensory stimulation and/or which brain regions need to be intact in order to benefit from multisensory stimulation. The discussed studies included patients mainly based on behavioral criteria and were not consistent in reporting their etiology (e.g., hemorrhagic or ischemic stroke), another factor that may well co-determine the effects of multisensory stimulation in rehabilitation. Future studies would benefit from standardized tasks and outcome measures. This could possibly contribute to establishing the degree of clinical relevance for observed outcome effects, quantified by, for example, the minimal clinically important difference (MCID). At the moment, scientific acceptance for the MCID is not yet achieved (Gatchel et al. 2010; King 2011). Establishing a quantification of clinical relevance would be valuable, as effects should not only have statistical significance, but also significance in improving the patients’ lives.

### Study Limitations

A limitation of the current review might be the incomplete retrieval of studies as the retrieval was limited to the selected keywords and databases. The selected inclusion and exclusion criteria resulted in inclusion of studies with a specific focus, while excluding studies on related subjects. Most noteworthy, this review selected studies on passive multisensory stimulation in sensory recovery after stroke. Obviously, stimulating motor recovery to prevent functional loss is very important as well (Nudo et al. 1996). Additionally, the type of studies included might only have been reported after a positive result. This results in a positive publication bias, which might have led to an underrepresentation of studies showing no beneficial effects of multisensory stimulation. A limitation regarding the included studies concerns the studies’ foci, which were mainly experimental. This resulted in an insufficient score on our quality assessment and restricts any conclusion on the clinical relevance of multisensory stimulation. Yet, the lack of studies focused on clinical application emphasizes the need for the implementation of proper RCTs.

## Conclusion

In conclusion, in recent years there has been a tremendous increase in fundamental cognitive neuroscience research on multisensory integration. In addition, a number of studies have reported promising results of multisensory stimulation in low-level as well as higher-level sensory impairments after stroke. Yet, as the quality of these studies was insufficient, at this moment it cannot be concluded that multisensory stimulation can be successfully applied as an effective intervention. It would be a valuable next step to continue this line of research with well-designed randomized controlled trials to examine whether and how multisensory stimulation can aid recovery after stroke.

## Abbreviations

ADL: Activities of Daily Living
fMRI: Functional Magnetic Resonance Imaging
MSI: Multisensory Integration
RCT: Randomized Controlled Trial
A: Auditory
AV: Audiovisual
d: Days
Fr: Frontal
h: Hours
LH: Left Hemisphere
m: Months
ms: Milliseconds
Oc: Occipital
P: Proprioceptive
Pa: Parietal
RH: Right Hemisphere
S: Somatosensory
SC: Subcortical
Sign.: Statistically Significant
Te: Temporal
V: Visual
v: Years
